# Glycemic control and antimicrobial prophylaxis audit in cardiac surgery in Uruguay

**DOI:** 10.1186/1753-6561-5-S6-O57

**Published:** 2011-06-29

**Authors:** H Albornoz, G Saona, I Wall, A Perna

**Affiliations:** 1 Fondo Nacional de Recursos. Uruguay, Montevideo, Uruguay

## Introduction / objectives

Glycemic control in cardiac surgery patients (pts) is one important factor that affect the risk of surgical site infection and mortality. Antimicrobial (ATM) profilaxis is complex process in this pts.

We evaluated the antimicrobial prophylaxis and glycemic control en ptes operate of cardiac surgery (CS) in Uruguay.

## Methods

An observational retrospective study of a population of cardiac surgery ptes operate between August 2006 and February 2007. A non proportional sample stratified by cardiac surgery center was selected and clinical records reviewed. Intra-operative and all values of the first 24 hours of plasmatic or percutaneous glucose concentration and data of antimicrobial prophylaxis (timing, ATMs, dose, intra-operative re-injection and duration) were recorded. Average peri-operative glycemia was estimated by area under the curve of glucose concentration-time.

## Results

610 (64.2 years, male 70.2%, bypass surgery 74%, diabetics 20.6%) patients were operated in the 5 centers of CS in Uruguay, the sample included 180 pts. All pts had at least one perioperative glycemic value. Average perioperative glycemia concentration was 1.77 g/L (diabetics 2.06, non diabetics 1.7 g/L). Ptes with postoperative infection had higher perioperative glycemia (all sites 1.89 vs 1.68 g/L, surgical site infections 1.9 vs 1.75 g/L, pÐ 0.001 and p=0.049, respectively). 43% of ptes received crystalline insulin infusion. 96.4% received one dose of ATM before the incision, 75.2% in the hour previous. 31.7% received intraoperative reinyection.

## Conclusion

Glycemic control and ATM prophylaxis need to be improved in Uruguay.

## Disclosure of interest

None declared.

